# A Novel Coordinated Motion Fusion-Based Walking-Aid Robot System

**DOI:** 10.3390/s18092761

**Published:** 2018-08-22

**Authors:** Wenxia Xu, Jian Huang, Lei Cheng

**Affiliations:** 1 Hubei Key Laboratory of Intelligent Robot, Wuhan Institute of Technology, Wuhan 430205, China; xuwenxia@wit.edu.cn; 2 Key Laboratory of Image Processing and Intelligent Control, School of Automation, Huazhong University of Science and Technology, Wuhan 430074, China; 3 School of Information Science and Engineering, Wuhan University of Science and Technology, Wuhan 430081, China; chenglei@wust.edu.cn

**Keywords:** walking-aid robot, multi-sensor fusion, human motion intention, coordinated motion

## Abstract

Human locomotion is a coordinated motion between the upper and lower limbs, which should be considered in terms of both the user’s normal walking state and abnormal walking state for a walking-aid robot system. Therefore, a novel coordinated motion fusion-based walking-aid robot system was proposed. To develop the accurate human motion intention (HMI) of such robots when the user is in normal walking state, force-sensing resistor (FSR) sensors and a laser range finder (LRF) are used to detect the two HMIs expressed by the user’s upper and lower limbs. Then, a fuzzy logic control (FLC)-Kalman filter (LF)-based coordinated motion fusion algorithm is proposed to synthesize these two segmental HMIs to obtain an accurate HMI. A support vector machine (SVM)-based fall detection algorithm is used to detect whether the user is going to fall and to distinguish the user’s falling mode when he/she is in an abnormal walking state. The experimental results verify the effectiveness of the proposed algorithms.

## 1. Introduction

An aging population dictates the need for elderly people to be able to live independently. Due to these individuals’ diminishing physical abilities, muscular strength and eyesight, the most important challenge in self-support is their ability to walk independently. Various locomotion assistive devices, such as wheelchairs, walkers and rehabilitation systems, have been developed and designed by researchers [1]. It is necessary to consider the maintenance and recovery of elders’ exercise capacity; however, even if these individuals are able to walk, the frequent use of a wheelchair may lead to atrophy of the lower limb muscles [2]. Therefore, robotic walking-aid systems, such as PAMM [3], RT walker [4], Care-O-bot 3 [5] and ORTW-II, have been proposed.

From the user’s perspective, a walking-aid robot should be compliant, meaning that the robot can comply with the interactive force between the robot and the user, as well as the user’s motion intention. A walking-aid robot user has limited self-mobility. To recover or maintain exercise capacity, the individual needs to use his/her remaining motion capability as much as possible. Therefore, understanding human motion intention (HMI) and generating appropriate and safe guidance commands for walking-aid robots is a primary issue.

Furthermore, human locomotion is characterized not only by leg movements, but also by a coordinated motion between the upper and lower limbs [6,7]. Monitoring other segments during human motion results in a more predictive and natural human-walker interaction, reaching a multi-modal interface.

There are several sensors used as the human-robot interface in some robot research, such as force sensors [8,9,10], touch screens [11], voice sensors [12], cameras [13], brain-computer interfaces [14], inertial sensors [15] and pressure sensors [16]. Force sensors are most commonly used in HMI estimations for walking-aid robots because they enable user-friendly HRIsby transforming interaction forces from the user to the desired robot motion velocity. It should be pointed out, however, that the force sensor-based HMI estimation method has some disadvantages. This method may result in a feeling of insecurity in the user when emergencies occur, for example in the event of a sudden fall. When the user falls with the robot, the interactive forces between the user and the robot will change drastically, resulting in an abrupt change in the robot’s moving velocity. On the other hand, there is an underlying proportional relationship between the HMI and the interactive forces in the force sensor-based HMI estimation method. To make a user feel comfortable and the robot compliant during operation of a walking-aid robot, the proportional relationship is represented by the corresponding coefficient in the impedance or admittance robot motion controller. If the coefficient is too large, the user will feel that the robot is too hard to “push”. Meanwhile, if the coefficient is too small, the user will feel that the robot is overly mobile, resulting in a feeling of insecurity or unsafety. Therefore, force sensor-based HMI estimation is not always a reliable method. The self-mobility of the walking-aid robot user should not feel diminished; the device should make the user feel as if he/she is handling a passive walking assistance apparatus.

The most important premise for a walking-aid robot is keeping the user safe, which means not only over the course of normal walking, but also when the user is in danger of unforeseen events, such as falling. Falling, which may occur due to physical or visual defects, is the most serious problem for walking-aid robot users, who are usually elderly or disabled people. Therefore, fall detection and prevention strategies are important, especially in the walking-aid robot system. Currently, there are few studies on fall detection and prevention strategies for walking-aid robots [9,10]. In the present paper, the user’s walking state is predicted by the coordinated motion between the upper and lower limbs. When the robot detects a potential fall, it applies an emergency brake to maintain the user’s safety.

In this paper, the main contributions include the following:

(1) We aim to investigate how to utilize the synergetic movements of the arms and legs to perceive more accurate HMI and a more compliant human-robot interface method in the normal walking state. According to the coordinated motion of the human-robot system, force sensors and an laser range finder (LRF) were used to detect the velocities of the human’s upper and lower limbs to estimate HMIs. The synergy of arm and leg movements will help the robot to perceive more accurate HMI and release a part of the user’s hand strength. Consequently, the user will feel that the robot is following rather than pushing him/her. Thus, the robot will better understand the user’s intention, and more effective guidance commands will be generated so that the walking-aid robot operates in a safe manner.
(2) Compared with the conventional force control methods (such as admittance control), the proposed coordinated motion-based motion control algorithm can detect the user’s abnormal gait in abnormal walking state, then remind the robot to react to prevent the user from falling.

The remainder of this paper is organized as follows. Section 2 introduces the related work. Section 3 describes the structure and working principle of our walking-aid robot, and the two HMI estimating algorithms for the upper and lower limbs. Section 4 proposes a novel coordinated motion fusion-based walking-aid robot system in the normal and abnormal walking state. Using multi-sensor fusion technology, the two HMIs are combined to obtain more accurate HMI and realize robot compliance motion control in normal walking state. Then, an SVM-based fall detection algorithm is used to detect the abnormal walking state. Section 5 verifies the proposed algorithms by various experiments. Furthermore, two comparative experiments are conducted to evaluate the proposed algorithms in Section 6. Finally, Section 7 draws the conclusions. The main symbols in our paper are presented in Table 1.

## 2. Related Work

To date, little research has considered the coordinated motion between the upper and lower limbs in robotic walking-aid systems. Stephenson [17] pointed out that high functioning stroke patients preserve the ability to coordinate the motion of upper and lower limbs and also suggested that the use of sliding handles in gait rehabilitation could be useful. Hirata [8] proposed a new walking support system based on cooperation between wearable-type and cane-type walking supports for hemiplegia patients; in this system, a wearable-type walking support device is used to detect the user’s leg motion, and a cane-type device is used to detect the user’s arm motion. Unlike [8], the force-sensing resistor (FSR) sensor-based human-robot interface is used to detect the user’s motion intention expressed by the upper limbs, and LRF is used to detect the user’s motion intention expressed by the lower limbs.

According to the different human-robot interfaces, the robotic walking-aid systems can be divided into two kinds: (1) contact-type sensor-based walking-aid systems; (2) non-contact-type sensor-based walking-aid systems. The contact-type sensor includes force sensors, joysticks, touch screens and voice activation systems. Considering the human-robot interactive force in the direction of the movement to estimate HMI, [10] presented a control system of an omnidirectional-type cane robot. Lu [18] designed novel low cost and highly reliable force-sensing handles for measuring the user’s applied force. Furthermore, he also designed an intelligent learning scheme to derive the proper driving force from the measured grip force to obtain HMI. Hans et al. proposed a robot with a touch screen as its interface [11]; the touch screen is the simplest HRI for a walking-aid robot. Since the input corresponds to the space shown on the screen, visual feedback can be displayed immediately. However, a touch screen would cause confusion for elderly users, increasing the likelihood of an accident. Kulyukin [12] proposed a voice activation system that translates two speech parameters (volume and pitch) to control the motions of robotic walkers. However, the drawbacks of this system were voice recognition and interference.

Non-contact-type sensor-based HMI estimation methods include the visual recognition method using cameras, the brain-computer interface-based HMI estimation method, the combination of a laser and inertial sensor and the combination of cameras and pressure sensors. Yu [13] used a camera to obtain user motion information, then studied the relationship of the sequence of human motions to recognize human intention. Carlson [14] proposed a brain-computer interface-based control algorithm for a robotic wheelchair. Cifuentes [15] proposed a human-robot interaction strategy, which was based on the acquisition of human gait parameters by means of data fusion from inertial measurement units and a laser range finder. A semi-automatic system for capturing footsteps was designed to gather a database comprised of more than 3500 footsteps from 55 persons [16]. Furthermore, human footsteps were captured by a camera and pressure sensors. Valado [19] designed a laser range finder and ultrasound sensors-based walker. In this human-robot system, the distance relationship between the robot and the user was considered as a formation. A laser range finder calculated the linear and angular walker’s velocity to keep the formation (distance and angle) in relation to the user. Our approach is the most similar to [19], but it improves upon the synergetic movements of the arms and legs to perceive more accurate HMI and a more compliant human-robot interface method. In this paper, we aim to investigate how to utilize the arms and legs’ synergetic movements of human intent estimation in walking-aid robot control. Both contact-type and non-contact-type sensors were used to obtain more accurate HMI; a laser ranger finder was used to detect the velocity of the human’s leg to estimate human motion intention. The synergies of arm and leg movements will help the robot obtain more accurate human motion intention, and release a part of the user’s hand strength; as a result, users will feel that the robot follows them rather than being pushed. Then, the robot can understand the user’s intention better and generate more appropriate guidance commands for the walking-aid robot in a safe manner.

To date, there has been little research on the fall detection and fall prevention strategies of walking-aid robots. Hirata [8] proposed a method for estimating the user states during the usage of the walker; this method supports the walking of the user based on the physical interaction between the user and the walker. A laser range finder and a tilting sensor were used to detect the user’s walking state (normal walking, upslope and emergency). If the distance between the user’s knee and robot was greater than a certain value, the robot stopped moving to prevent the user from falling. Then, Hirata [9] detected the joint position of the user’s lower limb to calculate the user’s COG in order to predict when the user was going to fall. However, the previous methods can only estimate the falls in the front and back direction, not the falls in the left and right direction. Huang [10] estimated the head position of a user by a round view camera and the relative distance between the user and robot by a laser range finder, then fused the two forms of data to predict whether the user was going to fall. Then, Huang [20] solved the fault detection and isolation (FDI) problem for the robotic assembly of electrical connectors in the framework of set-membership. In this paper, the user’s walking state was predicted by human motion intentions estimated by the movements of the upper and lower limbs, which were detected by force sensors and a laser range finder. When the robot detected a falling trend, it emergency braked to keep the user safe.

Recently, some machine learning approaches have been used in robotic systems. The reinforcement learning method was used in shared control for walking-aid robot motion control [21]. Meng [22] investigated an approach for robots to learn to adapt dance actions to human’s preferences through interaction and feedback. He [23] presented an unsupervised approach of integrating speech and visual information without using any prepared data. In this paper, an active-learning mechanism, called “desire for knowledge”, was used to let the robot select the object for which it possesses the least information for subsequent learning. Choi [24] proposed a mobile robot control method based on machine learning-neural network-based algorithms, which used only camera vision. Support vector machines (SVMs) are an effective machine learning method; they were originally designed for pattern recognition and classification tasks. Due to the good generalization property, SVMs have been successfully used in a wide variety of classification problems in robotics [25]. Compared to the conventional learning algorithm, SVM classifications may be more accurate than the widely-used alternatives such as classification by maximum likelihood, decision tree and neural network-based approaches [26]. Therefore, in this paper, the SVM method is used to classify the falling and the mode of falling.

## 3. Multi-Sensor-Based HMI Estimation Algorithms

### 3.1. Mechanism for the Walking-Aid Robot

**Figure 1:**
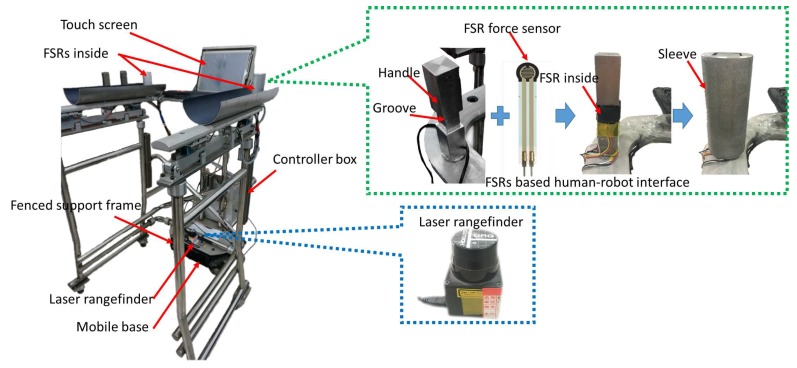
The walking-aid robot.

**Figure 2:**
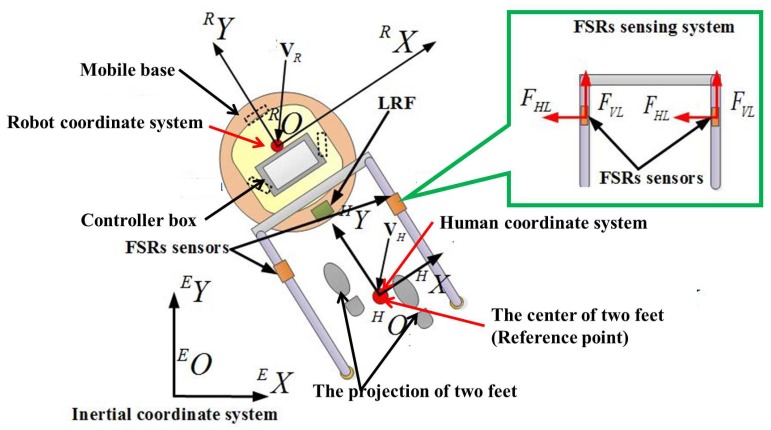
The walking-aid robot coordinate system (top view).

In this paper, the walking-aid robot we used (shown in Figure 1) consisted of an omni-directional mobile base, a fenced support frame, a motion controller and a multi-sensor sensing system. The multi-sensor sensing system was composed of a force-sensing resistor-(FSR)-based force-sensing system and an LRF sensor. The force-sensing system was a handle-sleeve type-based FSR pressure sensing device, as shown in Figure 1. FSR force sensors were installed in the four grooves of the inner handle, and the external sleeve was a circular sleeve. To increase the effective pressing effect of the force-sensing system, an FSR was packaged in two rubber sheets before installing the FSR sensors. Eight FSR sensors were used to measure the interactive forces between the robot and the user. Both forward and lateral forces could be obtained, as well as exerted rotation torque. One LRF was installed in the lower half of the robot to detect the user’s leg movements. The omnidirectional mobile base comprised three commercially available omni-wheels and actuators, which were specifically designed for the walking-aid robot. The coordinate systems are depicted in Figure 2.

### 3.2. FSR Sensor-Based HMI Estimation Algorithm

The arrangement of the FSR force sensors for estimating HMI is shown in Figure 1b. To replace an expensive six-axis force/torque sensor, FSR sensors were mounted on the four sides of each armrest, measuring the push/pull force of both hands.

We define {E} as the inertial frame, and {H} and {R} are local coordinate systems, which are fixed on the human and robot respectively (as shown in Figure 2). Consequently, the kinematics of the walking-aid robot can be represented by [27]. The intent force/moment is calculated by the following equations:

(1) $$\left\{\begin{array}{c} F_{Y}^{*}\;=\;F_{VL}+F_{VR}\;=\;\left[\left(F_{1}-F_{3}\right)+\left(F_{5}-F_{7}\right)\right]\\ F_{X}^{*}\;=\;F_{HL}+F_{HR}\;=\;\left[\left(F_{4}-F_{2}\right)+\left(F_{8}-F_{6}\right)\right]\\ M_{\theta }^{*}\;=\;K\left(F_{VR}-F_{VL}\right)=K\left(\left(F_{5}-F_{7}\right)-\left(F_{1}-F_{3}\right)\right) \end{array}\right.$$

where $F_{1}∼F_{8}$ are the force values detected by the eight FSR force sensors. $F_{X}^{*},F_{Y}^{*}$ and $M_{\theta }^{*}$ are the three-dimensional human intent force/torque. *K* is a proportionality coefficient.

Then, the desired walking velocity of the user $V_{H}={\left[{\dot{X}}_{H}\;{\dot{Y}}_{H}\;{\dot{\theta }}_{H}\right]}^{T}$ can be estimated as:

(2) $$\left\{\begin{array}{c} {\dot{X}}_{H}=\left\{\begin{array}{cc} K_{X}\left(\right|F_{X}^{*}\left|-F_{X0}\right)sgn\left(F_{X}^{*}\right), & |F_{X}^{*}|>F_{X0}\\ 0, & |F_{X}^{*}|\leq F_{X0} \end{array}\right.\\ {\dot{Y}}_{H}=\left\{\begin{array}{cc} K_{Y}K_{HR}\left(\right|F_{Y}^{*}\left|-F_{Y0}\right)sgn\left(F_{Y}^{*}\right), & |F_{Y}^{*}|>F_{Y0}\\ 0, & |F_{Y}^{*}|\leq F_{Y0} \end{array}\right.\\ {\dot{\theta }}_{H}=\left\{\begin{array}{cc} K_{\theta }\left(\right|M_{\theta }^{*}\left|-M_{\theta 0}\right)sgn\left(M_{\theta }^{*}\right), & |M_{\theta }^{*}|>M_{\theta 0}\\ 0, & |M_{\theta }^{*}|\leq M_{\theta 0} \end{array}\right. \end{array}\right.$$

where $F_{X0}$, $F_{Y0}$ and $M_{\theta 0}$ are the threshold values of the intention force/torque. $K_{X},K_{Y}$ and $K_{\theta }$ are the proportionality constants in each axis direction of the robot velocity. $K_{HR}$ is a switching value to restrain the relative distance between the human operator and the robot, which can be described by:

(3) $$K_{HR}=\left\{\begin{array}{cc} 1, & 0<D_{HR}<D_{MAX}\\ 0, & D_{HR}\geq D_{MAX} \end{array}\right.$$

Consequently, if the force vector is available, the user-desired motor velocity detected by the FSRs can be calculated according to Equations (1)–(3).

### 3.3. LRF-Based HMI Algorithm

The traditional force sensor-based HMI estimation algorithm has some disadvantages. If the user maintains a grip on the force sensor handle, when an emergency occurs, the interactive forces between the robot and the human are not zero, and the robot will continue to move; the user’s safety cannot be guaranteed by a moving robot. Therefore, the force sensor-based HMI estimation algorithm cannot be completely trusted, particularly when the user is not in the normal walking mode. Moreover, human gait is a coordinated motion between the upper and lower limbs. To obtain more accurate HMI, an LRF is used in this study to detect the velocity of the human’s legs in order to estimate HMI. Utilizing synergetic arm and leg movements and the user’s partial release of his/her hand grip helps the walking-aid robot to obtain more accurate HMI and improved motion control. Consequently, users will feel that the robot follows rather than pushes them.

Before estimating HMI by LRF, the human motion must be detected by an LRF and the human’s leg should be distinguished from the surroundings. Current environment sensing and detection systems mostly detect indoor (columns, corners, trash cans, doors, people, etc.) and outdoor (car parking poles, cars, etc.) structures. This geometric perception is important when making spatial inferences from which scene interpretation is achieved.

In the present research, for LRF detection, our choice of primitive feature for detection is the leg. Leg detection applications range from detecting human walking mode, to estimating HMI. It is typically assumed that a horizontal range scan is a collection of range measurements taken from a single robot position. When the robot is moving at high speed, this assumption is invalid. We used the rotation rate of the scanning device and the velocity of the robot to correct the errors of this assumption.

**Figure 3:**
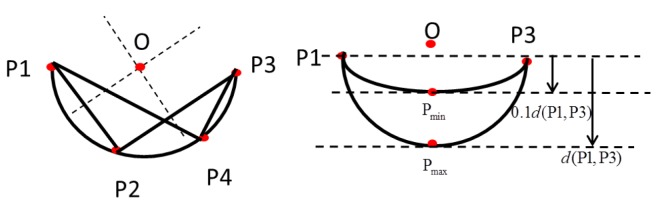
Parameters of the circle. P, point.

The whole LRF-based HMI estimation algorithm includes the following four steps:

1. Range segmentation:
  Due to the proximity of consecutive scan points probably belonging to the same object, range segmentation divides these consecutive scan points into a cluster. The segmentation method calculates the distance between two consecutive points because it is less than a given threshold. Isolated scan points are rejected.
2. Circle identification:
  We used the method in [28] to identify the circle. When a circle is identified, its center and radius need to be estimated. From analytic geometry, the three points on a unique circle P1, P2 and P3 constitute two secant lines (as shown in Figure 3). The first line denoted *a* passes through points P1 and P2, and the second line denoted *b* passes through points P2 and P3. The equations of these two lines are as follows:
  (4) $$y_{a}=m_{a}\left(x-x_{1}\right)+y_{1},m_{a}=\frac{y_{2}-y_{1}}{x_{2}-x_{1}}$$
  (5) $$y_{b}=m_{b}\left(x-x_{2}\right)+y_{2},m_{b}=\frac{y_{3}-y_{2}}{x_{3}-x_{2}}$$
  where $m_{a},m_{b}$ are the slopes of two secant lines.
  The center of the circle is the intersection of the two lines perpendicular to and passing through the midpoints of the secant line segments $\bar{P1P2}$ and $\bar{P2P3}$, as shown in Figure 3. The position of the center is as follows:
  (6) $$\left\{\begin{array}{c} x_{O}=\frac{m_{a}m_{b}\left(y_{1}-y_{3}\right)+m_{b}\left(x_{1}+x_{2}\right)-m_{a}\left(x_{2}+x_{3}\right)}{2(m_{b}-m_{a})}\\ y_{O}=\frac{m_{a}^{2}m_{b}\left(y_{1}-y_{3}\right)+m_{a}m_{b}\left(x_{1}+x_{2}\right)-m_{a}^{2}\left(x_{2}+x_{3}\right)}{2(m_{b}-m_{a})}-m_{a}x_{1}+y_{1} \end{array}\right.$$
  where line *a* passes through points P1 and P2, and line *b* passes through points P2 and P3.
  Since some circles are not human’s legs, a precondition is used to remove the segments that are not circles: the middle point of the segment must be inside an area delimited by two lines parallel to the extremes of the same segment, $0.1d\left(\bar{P1P2}\right)<d<d\left(\bar{P1P3}\right)$, as shown in Figure 3.
3. Leg detection:
  According to the inscribed angle theorem, if four consecutive points P1, P2, P3 and P4 are on the same circle, according to geometrical analysis, they have the same inscribed angles. Then:
  (7) ∠P1P2P3=∠P1P4P3
  After calculating the average of the inscribed angles of all points, if the standard deviation values are less than $8.6^{\circ }$ and the average values are between $90^{\circ }$ and $135^{\circ }$, the segment is classified as a circle. The procedure for detecting legs is an extension of circle detection. To identify a leg, the extra constraint of the distance between end-points falling within the range of expected leg diameters (0.1m–0.25 m) and the farthest distance between the LRF and a leg with a range of 0.3–1.2 m are proposed. Table 2 testifies the validity of the leg detection method when the user wears different clothes in different seasons. In the leg detection experiment, seven subjects wore their daily clothes and trousers in different seasons. All the success rates of the leg detection method in different seasons were 100%.
4. LRF-based HMI estimation:
  Walking is a process in which two legs move alternately, but using only the velocities of legs, it is difficult to express a human’s moving velocity and direction. Therefore, after detecting two leg positions relative to the LRF, we used the center position of the line segment, which consists of the positions of both legs to estimate the HMI:
  (8) $${\dot{X}}_{L}=\frac{{\dot{x}}_{l}+{\dot{x}}_{r}}{2}+{\dot{X}}_{R}$$
  (9) $${\dot{Y}}_{L}=\frac{{\dot{y}}_{l}+{\dot{y}}_{r}}{2}+{\dot{Y}}_{R}$$
  (10) $${\dot{\theta }}_{L}={\dot{\theta }}_{H}$$
  where $x_{l}$ and $y_{l}$ are the positions of the left leg and $x_{r}$ and $y_{r}$ are the positions of the right leg. ${\dot{X}}_{R}$ and ${\dot{Y}}_{R}$ are the actual velocities of the robot. Because it is difficult to obtain the intent angular velocity from the middle point of the two feet, in this paper, we formulated a design in which ${\dot{\theta }}_{L}$ is equal to ${\dot{\theta }}_{H}$. $V_{L}={\left[{\dot{X}}_{L}\;{\dot{Y}}_{L}\;{\dot{\theta }}_{L}\right]}^{T}$ are the desired velocities of the robot as detected by LRF.

The whole LRF-based HMI estimation algorithm is shown in Algorithm 1.


**Algorithm 1:**
    **Input:**
$x_{li}$ and $y_{li}$    **Output:**
$V_{L}$    1. Get each scan point position $x_{li}$ and $y_{li}$ by LRF.    2. Divide consecutive scan points into a cluster by range segmentation.    3. Identify the detected circle.    4. Calculate the center of the circles by Equations (4)–(6).     5. Identify the user’s legs from the detected circles.    6. Calculate the LRF-based HMI $V_{L}$ by Equations (8)–(10).

## 4. Coordinated Motion Fusion-Based Walking-Aid Robot System

Human locomotion is a synergy of arm and leg movements. To obtain a more predictive and natural human-walker interaction, a coordinated motion fusion-based walking-aid robot system is proposed in this paper. The proposed human-robot system can perceive more accurate HMI and release a part of the user’s hand strength. Meanwhile, the user will feel that the robot is following rather than pushing him/her. Furthermore, the robot will better understand the user’s intention, and more effective guidance commands will be generated so that the walking-aid robot operates in a safe manner.

Due to the different degrees of the upper and lower limb coordination and human-robot coordination, the user has different walking states when using the walking-aid robot [29]. Therefore, the robot can detect the user’s abnormal walking state by the coordinated motion of the user. In this article, we mainly categorize the walking states into two kinds: normal walking state; abnormal state (emergency state). In the normal walking state, a coordinated motion fusion-based compliance control algorithm is proposed. Furthermore, the coordinated motion-based fall detection algorithm is proposed to detect the user’s abnormal walking state.

### 4.1. Coordinated Motion Fusion-Based Compliance Control Algorithm in the Normal Walking State

In the normal walking state, compliance is the most important property for a human-robot system. Compliant motion allows a robot or an object held by a robot to comply with the interaction forces generated by its contact with the objects in an environment [30]. As a human-machine interface, a traditional force sensor is applied for various compliance motion controls for the walking-aid robot. Due to a user’s physical condition or external distractions in the environment, a user may fall when operating a walking-aid robot. Therefore, if a force sensor is applied as the one and only human-machine interface, misoperation may occur due to the user pressing force sensors or some other reasons. Therefore, force sensor-based HMI estimation methods are not entirely trustworthy. Consequently, in this paper, FSR sensors and an LRF were used to estimate the user’s intentions; multi-sensor fusion technology was applied to utilize the synergetic movements of the arms and legs for a coordinated motion fusion algorithm. The synergy of arm and leg movements facilitates the robot’s obtainment of more accurate HMI. The walking-aid robot will comply with the user’s motion intention and realize compliant motion control.

#### 4.1.1. Kalman Filter-Based Coordinated Motion Fusion Algorithm

Currently, due to requirements of comprehensive and exact information, data fusion methods are widely used in various robot measurement systems. Multi-sensor data fusion is a recent trend in sensor technology. It is a technology that explores the abundant information in nature by different homogenous or heterogeneous sensors and fuses them for high-level decision making. It mainly combines multi-sensor information, which is redundant and/or complementary in space or time, to obtain a uniform description or understand a measured object according to a certain criterion. The Kalman filter has good performance in dynamic sensor information fusing in real time. In this section, the Kalman filter algorithm is used to fuse the two HMIs detected by the FSR sensors and LRF. When a user turns to the left or right with a walking-aid robot, typically, he/she will not move his/her leg, but spin the body to the left or right. Therefore, in the multi-sensor fusion algorithm, we do not consider the rotational speed of humans. The final human intent rotational speed is estimated by FSR sensors, as introduced in Section 3. The HMI Estimation Algorithm I (FSR-based HMI estimation) was introduced in Section 3.2, and HMI Algorithm II (LRF-based HMI estimation) was introduced in Section 3.3. $V_{H}$ and $V_{L}$ are the human intent motion velocities estimated by FSR sensors and LRF, respectively, and they are the input of the Kalman filter. Next, the filtered human intent motion velocities $V_{F}=\left[{\dot{X}}_{F}\;{\dot{Y}}_{F}\;{\dot{\theta }}_{F}\right]$ are sent to the motor to effect motion.

The equations for the Kalman filter are based on [31] and are described below. A Kalman filter works like a feedback controller. The filter estimates the next state of the signal (predict) and then obtains feedback in the form of noisy measurements to modify the predicted state (correct). The defined state variables are as follows:

(11) $$X={\left[{\dot{X}}_{F}\;{\dot{Y}}_{F}\;{\dot{\theta }}_{F}\;{\ddot{X}}_{F}\;{\ddot{Y}}_{F}\;{\ddot{\theta }}_{F}\right]}^{T}$$

(12) $$Z={\left[{\dot{X}}_{H}\;{\dot{Y}}_{H}\;{\dot{\theta }}_{H}\;{\dot{X}}_{L}\;{\dot{Y}}_{L}\;{\dot{\theta }}_{L}\right]}^{T}$$

where $V_{H}=\left[{\dot{X}}_{H}\;{\dot{Y}}_{H}\;{\dot{\theta }}_{H}\right]$ is the motion intention estimated by HMI Estimation Algorithm I and $V_{L}=\left[{\dot{X}}_{L}\;{\dot{Y}}_{L}\;{\dot{\theta }}_{L}\right]$ is the motion intention estimated by HMI Estimation Algorithm II. $V_{F}=\left[{\dot{X}}_{F}\;{\dot{Y}}_{F}\;{\dot{\theta }}_{F}\right]$ is the fused HMI motion velocity. Then, the state-space equations are:

(13) $$\left\{\begin{array}{c} X_{i}=AX_{i-1}+w_{i}\\ Z_{i}=HX_{i}+v_{i} \end{array}\right.$$

(14) $$A=\left[\begin{array}{cccccc} 1 & 0 & 0 & △t & 0 & 0\\ 0 & 1 & 0 & 0 & △t & 0\\ 0 & 0 & 1 & 0 & 0 & △t\\ 0 & 0 & 0 & 1 & 0 & 0\\ 0 & 0 & 0 & 0 & 1 & 0\\ 0 & 0 & 0 & 0 & 0 & 1 \end{array}\right],H=\left[\begin{array}{cccccc} a & 0 & 0 & 0 & 0 & 0\\ 0 & a & 0 & 0 & 0 & 0\\ 0 & 0 & 1 & 0 & 0 & 0\\ b & 0 & 0 & 0 & 0 & 0\\ 0 & b & 0 & 0 & 0 & 0\\ 0 & 0 & 1 & 0 & 0 & 0 \end{array}\right]$$

where *i* is the sampling time and △t is the sampling time interval. *A* is the parameter matrix. *H* is the measurement system parameter, which denotes the degree of confidence of the two HMI estimation algorithms. *a* is the confidence variable of HMI algorithms, and b=1−a. *w* and *v* are the process and measurement noises, respectively, and are both Gaussian white noise.

According to the system model, the equation for the “predict” stage can be put into the general form:

(15) $$X_{i+1}^{*}=AX_{i}$$

(16) $$P_{i+1}^{*}=AP_{i}A^{T}+Q$$

where *Q* is the process covariance and $P_{i+1}^{*}$ is the a priori estimated error covariance.

According to the predicted system state and the observed system state, the “correct” stage can be presented as:

(17) $$K_{i+1}=P_{i+1}^{*}H^{T}{\left(HP_{i+1}^{*}H^{T}+R\right)}^{-1}$$

(18) $$X_{i+1}=X_{i+1}^{*}+K_{i+1}\left(Z_{i+1}-HX_{i+1}^{*}\right)$$

(19) $$P_{i+1}=\left(I-K_{i+1}H\right)P_{i+1}^{*}$$

where *R* is the measurement noise covariance and *K* is the system gain. Then, on the basis of Equations (15)–(19), we can obtain the fused HMI.

#### 4.1.2. Fuzzy Logic Adaptive System

**Figure 4:**
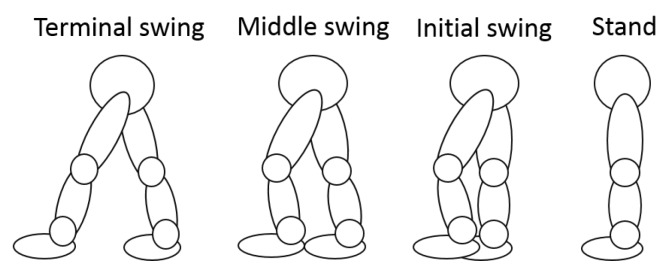
Parameters of the circle.

In the Kalman filter-based coordinated motion fusion system described above, matrix *H* denotes the degree of confidence of the two HMI estimation algorithms. *a* is the confidence variable of HMI algorithms. If a = 0, the robot will have greater trust in the human intention motion velocity, which is estimated by HMI Estimation Algorithm II. Conversely, if a = 1, more trust is attributed to the FSR-based HMI velocity. Due to the different rhythms of the two HMI velocities, the robot should assign different degrees of confidence to the two HMI velocities in the different walking processes (initial swing phase, middle swing phase and terminal swing phase, as shown in Figure 4):

1. Initial swing: At the beginning of a stride, FSR-based HMI velocities are smaller than LRF-based HMI velocities. When an individual uses a robot, he/she will sense that the robot is heavy and must exert effort to push the robot. If the robot assigns more trust to the LRF-based HMI velocities, it will have a faster starting speed. Therefore, the user can apply less strength to manipulate the robot and feel more comfortable.
2. Middle swing: When the user is in the middle swing phase, both of the HMI velocities have reached their peak values and the robot trusts both of them.
3. Terminal swing: In the terminal swing phase, the LRF-based HMI velocities will decrease rapidly, and the FSR-based HMI velocities will remain unchanged. At this time, due to safety requirements, the robot should assign more trust to the FSR-based HMI velocities.

The fuzzy logic method is widely used in robotic systems [32]. Then, fuzzy logic is used to adjust the confidence variable *a* of HMI algorithms online in the Kalman filter-based coordinated motion fusion algorithm in this section.

**Figure 5:**
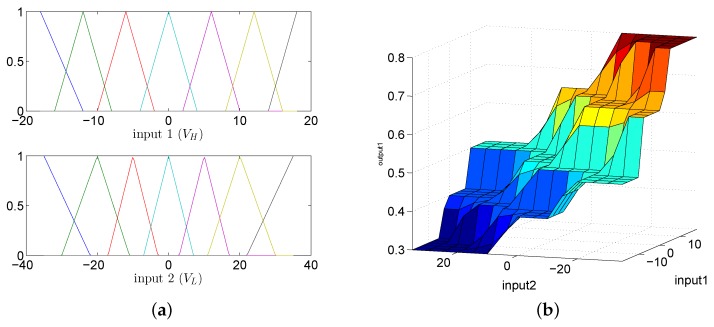
The fuzzy logic controller (Input 1 is $V_{H}$; Input 2 is $V_{L}$). (**a**) The input membership functions. (**b**) The fuzzy logic rules.

The FLC has two inputs: $V_{H}$ (Input 1) and $V_{L}$ (Input 2); one output: *a* (Output 1); and uses 49 rules. The linguistic variables for $V_{H}$ and $V_{L}$ are negative big (NB), negative medium (NM), negative small (NS), zero (Z), positive small (PS), positive medium (PM) and positive big (PB). The linguistic variables for *a* are very small (VS), small (S), medium (M), small big (SB) and very big (MB), and they are quantized into five levels represented by: 0.4, 0.5, 0.6, 0.7 and 0.8. The membership functions of these two inputs are shown in Figure 5a. The fuzzy logic rules are shown in Figure 5b and Table 1.

**Figure 6:**
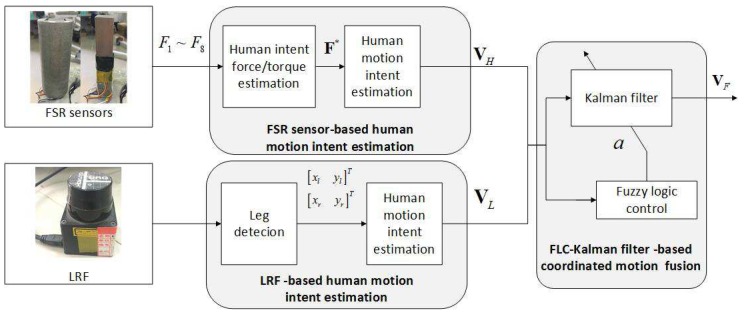
The fuzzy logic control (FLC)-Kalman filter-based coordinated motion fusion algorithm for the walking-aid robot.

The proposed FLC-Kalman filter-based coordinated motion fusion algorithm for walking-aid robot compliance control in normal walking state is shown in Algorithm 2 and Figure 6.


**Algorithm 2:**
    **Input:**
$V_{H}$, $V_{L}$    **Output:**
$V_{F}$    1. Get $V_{H}$ and $V_{L}$.    2. Calculate the membership functions of $V_{H}$ and $V_{L}$    3. Calculate the output *a* (the confidence variable of HMI algorithms) by the fuzzy logic rules in Table 3.    4. Get the state variables by Equations (11) and (12).    5. Calculate the “predict” stage of the Kalman filter.    6. Calculate the “correct” stage of the Kalman filter according to the confidence variable *a*.    7. Get the output $V_{F}$ of the Kalman filter.

### 4.2. Coordinated Motion-Based Fall Detection Algorithm in the Abnormal Walking State

Safety is the precondition for a human-robot system; therefore, a fall detection algorithm is proposed in this section. Falling can be detected according to the coordinated motion between the user’s upper and lower limbs, as well. The walking-aid robot we used has fence-type structural support and an arm fixer, so the probability of falling is low. When humans use the robot, possible falls include (i) falling forward, (ii) falling to the left, and (iii) falling to the right.

Based on the possible types of fall, the next task in our fall detection algorithm is estimating the possible falling modes of the user according to the coordinated motion between the upper and lower limbs. Compared with other traditional learning algorithms, SVMs have significant advantages in small sample learning, and their optimal classification hyperplane depends on only a few key samples, namely the support vector. Therefore, in the next step, we use SVM to learn the real-time user’s falling mode to classify new intent velocity data for upper and lower limbs based on the learned model. Due to the computationally-intensive training required for SVMs, the data training was performed offline. New data were classified online, as this step is fast.

SVMs belong to the family of kernel methods [33], which are currently extremely popular in the field of machine learning. The main idea of SVM is to construct a separating hyperplane between two classes of points, such that the margin between the hyperplane and the points closest to it becomes maximal. A linear SVM classification approach can be achieved by looking for an optimal hyperplane that separates the two classes in input data X to maximize the separating margin. In a nonlinear case, it can be achieved by first mapping the original data to some high-dimensional feature space. In a nonlinear method, then, the linearly non-separable data are first mapped with a kernel method in a higher dimensional feature space, which defines a dot product between points in the feature space. It is also possible to allow for a small number of training errors by means of a so-called soft margin parameter that regularizes the trade-off between maximizing the margin and minimizing the training error.

SVMs have an extremely good effect on binary classification questions. When an SVM is used in a multi-class classification problem, there are two possible solutions: (1) one-against-one or (2) one-against-all. In the one-against-all solution, the system is trained with each class classified against the samples of all the other classes [34]. The one-against-one method, in which classes are classified into pairs, has higher classification accuracy and is widely used. In this paper, the one-against-one solution is applied to predict the user’s falling mode.

**Figure 7:**
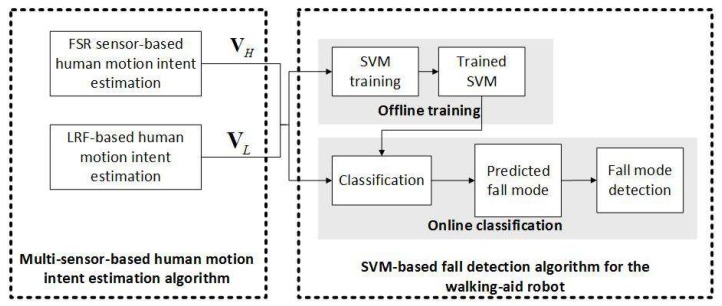
The fall detection algorithm for the walking-aid robot.

We employed (x,y) as the training data for the SVM, where $x=({\dot{X}}_{F},\;{\dot{Y}}_{F},\;{\dot{\theta }}_{F},\;{\dot{X}}_{L},\;{\dot{Y}}_{L},\;{\dot{\theta }}_{L})$ and y={1,2,3} represent the user’s falling mode. The SVM for the fall detection algorithm for the walking-aid robot is shown in Figure 7. We used LIBSVM software for SVM implementation.

## 5. Experiment

### 5.1. Coordinated Motion Fusion-Based Compliance Control Experiments in the Normal Walking State

**Figure 8:**
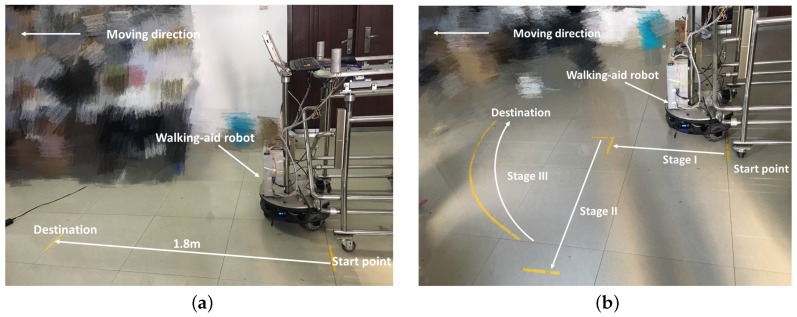
The two compliance control experiments. (**a**) The user goes straight with the robot. The white arrow line indicates the robot’s moving direction. (**b**) User goes straight (Stage I) → goes to the left (Stage II) → follows the curve (Stage III). The white arrow line indicates the robot’s moving direction.

First, two compliance control experiments were conducted to test the proposed coordinated motion fusion-based compliance control algorithm from Section 4.1, as shown in Figure 8. In this figure, the yellow lines are the start point and destination, and the white arrow lines represent the path of the robot. The distance between the start point and destination is 1.8 m. In Compliance Control Experiment I, the user goes straight with the robot, as shown in Figure 8a. Compliance Control Experiment II consists of three walking mode. Firstly, the user goes straight (Stage I), then goes to the left (Stage II) and finally follows the curve (Stage III), as shown in Figure 8b.

**Figure 9:**
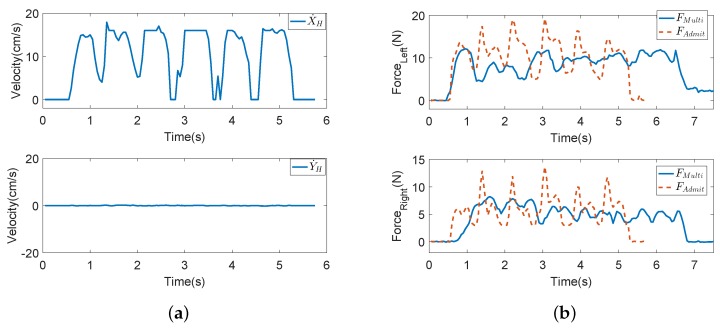
The results of Compliance Control Experiment I. In this experiment, the user goes straight with the robot. (**a**) The velocities in the X-axis. (**b**) The velocities in the Y-axis.

**Figure 10:**
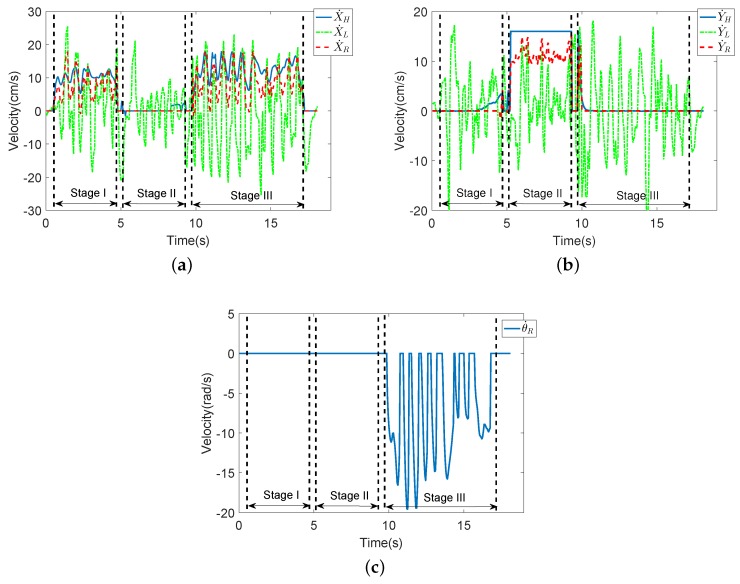
The results of Compliance Control Experiment II. In this experiment, the user goes straight (Stage I) → goes to the left (Stage II) → follows the curve (Stage III). (**a**) The velocities in the X-axis. (**b**) The velocities in the Y-axis. (**c**) The angular velocity of the robot.

The experimental results of the compliance experiments are shown in Figure 9 and Figure 10. In Figure 9, the blue line is the HMI velocity estimated via the user’s upper limb, the green line is the HMI velocity estimated via the user’s lower limb and the red dotted line is the actual motion velocity fused by the FLC-Kalman filter-based coordinated motion fusion algorithm proposed in Section 4.1. Because the user moves forward, the robot’s horizontal velocity and rotational angular velocity are zero in Figure 9b. According to the experimental results for human walking velocity in the literature, it is known that walking is an action derived by alternate swinging of the legs. This means that a human’s walking velocity is similar to a sine curve, which consists of a series of crests and troughs [35]. In Figure 9a, compared with the estimated intent velocities of the upper and lower limbs, the fused actual robot velocity contains more obvious and logical crests and troughs, which agrees with the description about human walking velocity. In other words, the fused motion velocity is closer to the human walking pattern. The user will feel that the robot follows the user rather than pushing the robot to walk. Therefore, a user is able to manipulate the robot more compliantly and comfortably.

Figure 10 shows the experimental results for several walking modes. In Experiment II, the user goes straight → goes to the left → follows the curve. In this figure, it can be seen that the fused robot velocities are similar to actual human walking velocities. In conclusion, the proposed coordinated motion fusion-based compliance motion control algorithm can obtain better compliance and a more accurate HMI velocity.

The experiment result of Compliance II: In this experiment, the user goes straight;→ goes to the left → follows the curve.

**Figure 11:**
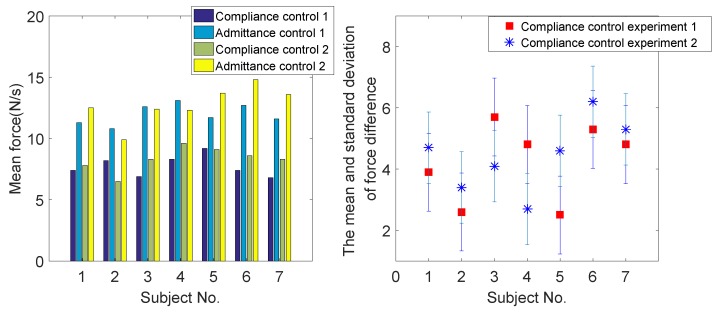
The mean interactive force and the standard deviation of the interactive force difference of seven subjects. The red box is the standard deviation of interactive force in Compliance Control Experiment I. The blue ∗ is the standard deviation of interactive force in Compliance Control Experiment II.

As shown in Figure 11, the mean interactive force between the robot and human, with the coordinated motion fusion-based compliance motion control algorithm, is smaller than with the conventional admittance control algorithm. The experimental results ensure the feasibility of the proposed coordinated motion fusion-based compliance motion control algorithm.

### 5.2. Coordinated Motion-Based Fall Detection Experiments in the Abnormal Walking State

**Figure 12:**
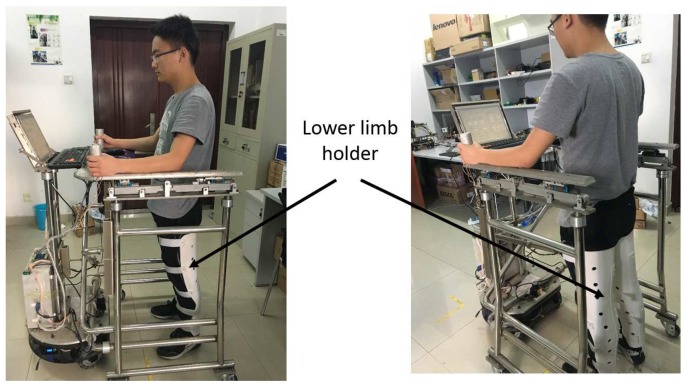
A user in a lower limb holder.

Before the experiments, to obtain human intent velocities for different subjects in different falling modes, offline human intent velocity data needed to be collected. In the process of data collection, a lower limb holder was used to decrease user motion and imitate a user with disabled motion ability, as shown in Figure 12. Seven subjects voluntarily took part in the experiments. The physical parameters of the subjects are shown in Table 4. Each subject was asked to fall forward, fall to the left and fall to the right 20 times, respectively, while using the walking-aid robot.

**Figure 13:**
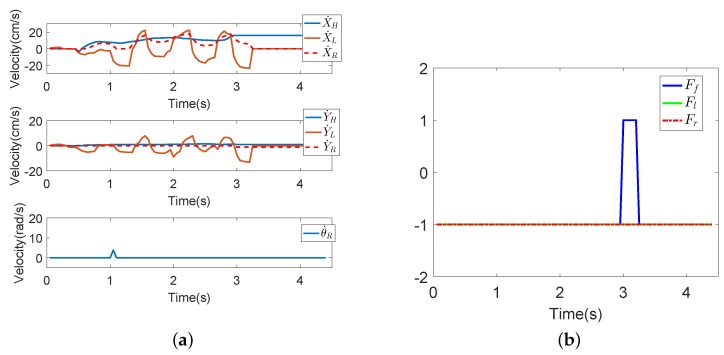

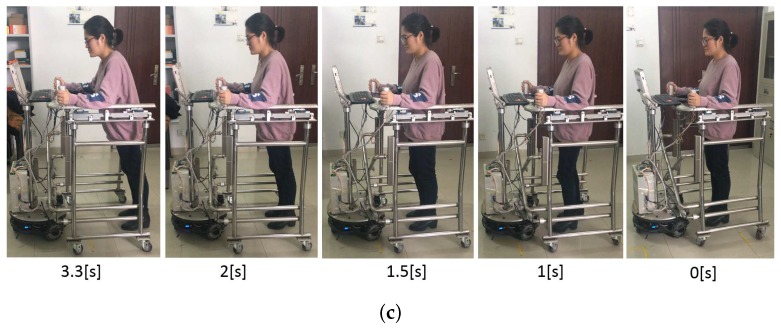
The results of the falling forward experiment. (**a**) The HMI velocities estimated by HMI Estimation Algorithms I and II and the actual robot moving velocities by the KF-based coordinated motion fusion algorithm when the subject falls forward. (**b**) The result of fall mode detection when the subject falls forward. (**c**) The process of falling forward.

**Figure 14:**
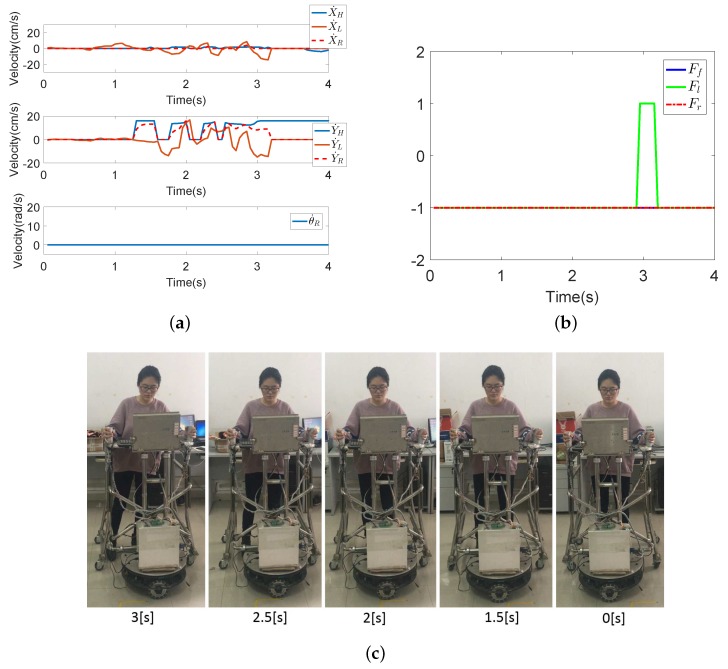
The results of the falling to the left experiment. (**a**) The HMI velocities estimated by HMI Estimation Algorithms I and II and the actual robot moving velocities by the KF-based coordinated motion fusion algorithm when the subject falls to the left. (**b**) The result of fall mode detection when the subject falls to the left. (**c**) The process of falling to the left.

**Figure 15:**
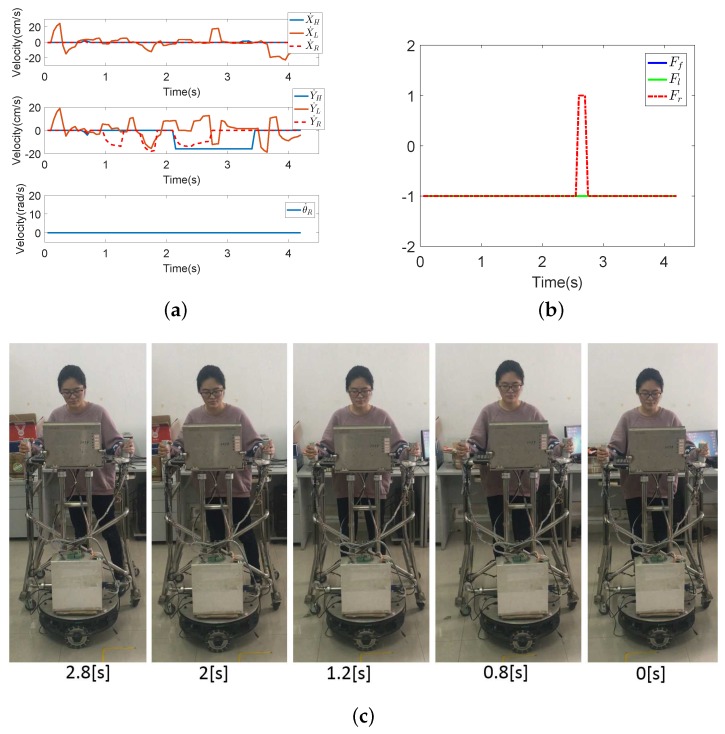
The results of the falling to the right experiment. (**a**) The HMI velocities estimated by HMI Estimation Algorithms I and II and the actual robot moving velocities by the KF-based coordinated motion fusion algorithm when the subject falls to the right. (**b**) The result of fall mode detection when the subject falls to the right. (**c**) The process of falling to the right.

Seven subjects took part in fall detection experiments in the abnormal walking mode. Each subject, respectively, fell forward, fell to the left and fell to the right 20 times, respectively. The proposed fall detection algorithm for a walking-aid robot presented in Section 4.2 was implemented in these experiments. When the robot detects that the user is going to fall, the robot stops immediately for the user’s safety. Figure 13, Figure 14 and Figure 15 show the results of the three fall detection experiments. In these figures, $V_{H}=\left({\dot{X}}_{H},\;{\dot{Y}}_{H},\;{\dot{\theta }}_{H}\right)$ are the HMI velocities estimated by HMI Estimation Algorithm I, and $V_{L}=\left({\dot{X}}_{L},\;{\dot{Y}}_{L}\right)$ are the HMI velocities estimated by HMI Estimation Algorithm II. $F_{f}$, $F_{l}$ and $F_{r}$ are the flags of the three fall modes. It can be seen that the proposed fall detection algorithm successfully detects the falling mode.

Figure 13 shows the results of Falling Mode I (fall forward) experiment. When the user is falling forward, he/she leans forward, shown in Figure 13c. As the user’s hands push the robot, ${\dot{X}}_{F}$ reaches the maximum value of 16. As the lower limbs of the user do not move, ${\dot{X}}_{L}$ decreases rapidly at the same time. Then, in Figure 13b, $F_{f}$ increases to one in 3 s; that is to say, the robot detects that the user is going to fall forward.

Figure 14 shows the result of Falling Mode II (fall to left) experiment. When the user is in Falling Mode II, the user leans to the left (as shown in Figure 14c). Then, the interactive horizontal force increases, and ${\dot{Y}}_{F}$ reaches the maximum value of 16. At this time, the user’s lower limbs do not move, but the robot continues to move to the left. As a result, ${\dot{Y}}_{L}$ decreases rapidly at the same time. In Figure 14b, $F_{l}$ increases to one in almost 3 s, meaning that the robot detects that the user is going to fall to the left.

Figure 15 shows the result of Falling Mode III (fall to the right) experiment. Contrary to Falling Mode II, the user leans to the right. In this falling mode, the interaction horizontal force increases. Therefore, ${\dot{Y}}_{F}$ reaches a negative maximum value of −16. As the lower limbs of the user do not move and the robot continues to move to the right, ${\dot{Y}}_{L}$ increases rapidly at the same time. In Figure 15b, $F_{r}$ increases to one in almost 2.6 s, meaning that the robot detects that the user is going to fall to the right.

**Figure 16:**
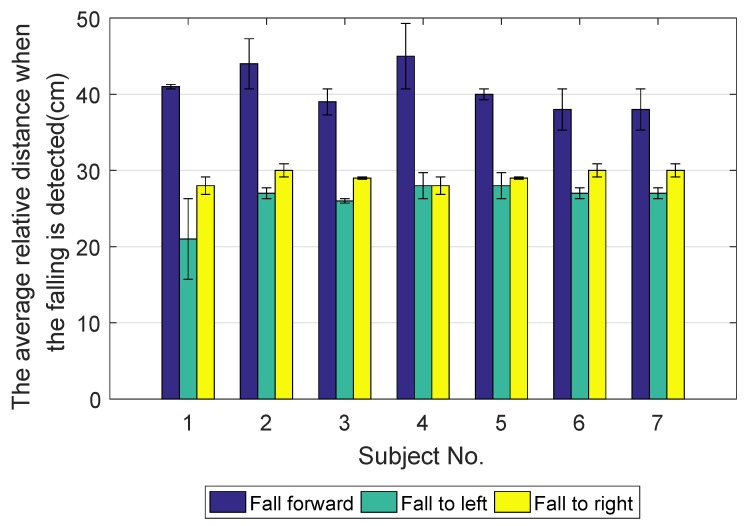
The mean average relative distance when falling is detected (error bar) in seven subjects.

Figure 16 shows the mean average relative distance when falling is detected (error bar) in seven subjects. The blue error bar is the mean average relative distance when the user falls forward. The green error bar is the mean average relative distance when the user falls to the left. The yellow error bar is the mean average relative distance when the user falls to the right.

## 6. Comparative Experiment

### 6.1. Comparative Compliance Control Experiment in Normal Walking State

**Figure 17:**
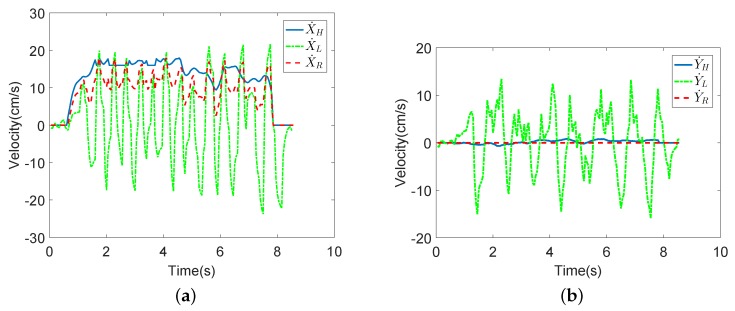
The results of the comparative compliance control experiment. (**a**) Admittance control experiment. In this experiment, the user goes straight with the robot. (**b**) Comparison of the interactive forces of the proposed coordinated motion fusion-based compliance control algorithm and admittance control.

In this section, a comparative experiment is conducted to verify the effectiveness of the proposed coordinated motion fusion-based compliance control algorithm. Admittance control and impedance control are the most common compliance control algorithms in walking-aid robot motion control. Accordingly, admittance control is applied in our walking-aid robot for comparison with the proposed coordinated motion fusion-based compliance control algorithm. In the admittance control experiment, Subject 1 goes straight with the same velocity and in the same experimental environment as in Compliance Experiment I. Figure 17a is the robot motion velocity of admittance control, and the blue line is the HMI velocity estimated by HMI Estimation Algorithm I. Figure 17b is the interactive force comparative result between the coordinated motion fusion-based compliance control and the admittance control. In this figure, the blue line is the interactive force of the coordinated motion fusion-based compliance control algorithm, and the red dotted line is the interactive force of the admittance control. It can be seen from Figure 17 that the interactive force of the coordinated motion fusion-based compliance control is smaller and smoother than the admittance control. Considering the results of these two figures, it can be determined that the user can apply less force to manipulate the walking-aid robot with the proposed coordinated motion fusion-based compliance control algorithm, and the motion is more compliant and comfortable.

### 6.2. Comparative Fall Detection Experiments in the Abnormal Walking State

To verify the validity of the proposed fall detection algorithm, we compared it with the fall detection method proposed by Huang [36]. In these comparative experiments, we conducted a wearable sensor-based fall detection experiment on Subject 1 in the same environment used in the previously conducted experiments. The details of the wearable sensor-based fall detection method for the walking-aid robot can be seen in [36]. In this algorithm, the subject applies wearable sensors to detect the distance between his center of pressure (COP) and the midpoint of his two feet, which is assumed to be a significant feature in the detection of fall events. Then, the Dubois possibility theory is applied to describe the membership function of a ‘normal walking’ state. A threshold-based fall detection approach is obtained from online evaluation of the subject’s walking status.

**Figure 18:**
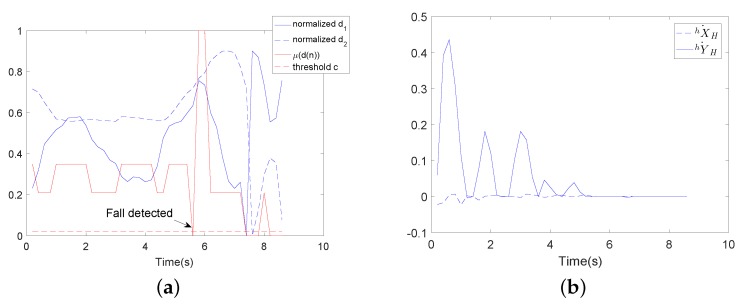
Wearable sensor-based user fall detection experiment using the walking-aid robot (falling to the left). (**a**) Feature d1(n) is the distance between the center of pressure (COP) and the midpoint of the user’s two feet. Feature d2(n) is the height of the user’s waist. μ(d(n)) is the membership degree value. The threshold value is 0.2. (**b**) The moving velocities of the robot.

**Figure 19:**
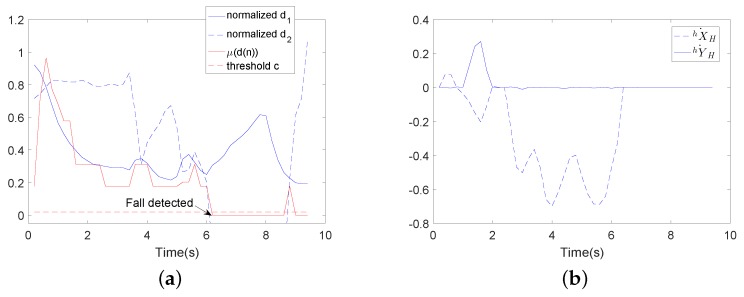
Wearable sensor-based user fall detection experiment using the walking-aid robot (falling down). (**a**) Feature d1(n) is the distance between the center of pressure (COP) and the midpoint of the user’s two feet. Feature d2(n) is the height of the user’s waist. μ(d(n)) is the membership degree value. The threshold value is 0.2. (**b**) The moving velocities of the robot.

Figure 18 and Figure 19 show the comparative experimental results for the fall detection method. In these figures, $d_{1}$ (the distance between the COP and the midpoint of the user’s two feet) and $d_{2}$ (the height of the user’s waist) are the significant features detected by the wearable sensors. μ(d(n)) is the membership degree value of significant features, and c is the threshold value for fall detection. In the experiments, the threshold value c=0.02. If μ(d(n))<c, the user is predicted to have the tendency to fall. As in Figure 18, μ(d(n))<c in about 5.8 s, then falling is detected. In Figure 19, μ(d(n))<c in about 6 s, then falling is detected. It can be seen that this method can successfully detect the tendency to fall, but it cannot detect the fall mode (falling forward, falling to the left or falling to the right). Before the comparative experiment, the subject first needed to wear the wearable sensors. The way these sensors are worn influences the accuracy rate of the fall detection method. However, the proposed fall detection algorithm in this paper used data detected by an LRF. The LRF’s degree of reliability is greater than that of wearable sensors. In our proposed fall detection method, there is no need for extra wearable sensors. The fall detection method used in the comparative experiment needs to determine a specific walking state. Such a determination is not necessary with our proposed algorithm.

Table 5 is the comparative result of the average relative distances when falling is detected. In the table, the second column is the longitudinal relative distance between the user and the robot when the user falls forward; the third and forth columns are the horizontal relative distances between the initial position and the fall detection position when the user falls to the left and right. It can be seen that the average relative distances of the proposed fall detection algorithm are smaller than the comparative fall detection algorithm. This means that the proposed fall detection algorithm can detect falling more quickly than the comparative fall detection algorithm.

## 7. Conclusions

This paper proposed a novel coordinated motion fusion-based walking-aid robot operated in both normal and abnormal walking modes. Human locomotion is not only characterized by leg movements, but also by coordinated motion between the upper and lower limbs. With the user in the normal walking mode, an FLC-KF-based coordinated motion fusion algorithm was proposed to fuse the two HMIs expressed by the motions of the upper and lower limbs. Moreover, a walking-aid robot should maintain the safety of the user, not only in normal walking mode, but also in abnormal walking mode. Therefore, we used an SVM-based fall detection algorithm for a walking-aid robot in abnormal walking mode to detect a user’s falling mode. If the robot detected that the user would fall, the robot stopped moving immediately to keep the user safe. Experiments were conducted to verify the effectiveness of the proposed walking-aid robot system in normal and abnormal walking states.

There were some limitations to our motion control system. If the robot stops moving to prevent the user from falling, the emergency stop cannot ensure complete user safety. Because the user is in an abnormal walking mode when the robot stops moving, due to the user’s physical disability or decreased mobility, the user is likely to fall again after the emergency stop. In future work, we plan to conduct detailed research on fall prevention motion control for the three falling modes (falling forward, falling to the left, and falling to the right).

## Figures and Tables

**Table 1:** The symbols in our paper. FSR, force-sensing resistor; LRF, laser range finder; HMI, human motion intention.

$F_{1}∼F_{8}$	The force values of the eight FSRs
$F_{X}^{*},F_{Y}^{*},M_{\theta }^{*}$	The human intent force and torque
$V_{H}={\left[{\dot{X}}_{H}\;{\dot{Y}}_{H}\;{\dot{\theta }}_{H}\right]}^{T}$	The desired walking velocity of the user
$F_{X0},F_{Y0},M_{\theta 0}$	The threshold values of the intention force/torque
$K_{X},K_{Y},K_{\theta }$	The proportionality constants of the robot velocity
$K_{HR}$	A switching value to restrain the relative distance between the user and the robot
$D_{MAX}$	The threshold values of max relative distance between the human and the robot
$x_{O},y_{O}$	The center of a circle.
$m_{a},m_{b}$	The slopes of Lines a and b.
$x_{l},y_{l},x_{r},y_{r}$	The positions of the left leg and right leg.
$x_{li},y_{li}$	Each scan point position of the LRF.
$V_{L}={\left[{\dot{X}}_{L}\;{\dot{Y}}_{L}\;{\dot{\theta }}_{L}\right]}^{T}$	The desired velocities of the robot as detected by the LRF
$V_{F}=\left[{\dot{X}}_{F}\;{\dot{Y}}_{F}\;{\dot{\theta }}_{F}\right]$	The fused HMI motion velocity

**Table 2:** The success rate of the leg detection method in different seasons.

Subject No.	Male/Female	Summer		Winter	
		Shorts/Shirt	Success Rate	Tight Trousers/Loose Trousers	Success Rate
1	Female	Shirt	100%	Tight trousers	100%
2	Female	Shirt	100%	Loose trousers	100%
3	Male	Shorts	100%	Tight trousers	100%
4	Male	Shorts	100%	Loose trousers	100%
5	Male	Shorts	100%	Loose trousers	100%
6	Female	Shirt	100%	Tight trousers	100%
7	Female	Shirt	100%	Loose trousers	100%

**Table 3:** Rules table for a. negative big (NB), negative medium (NM), negative small (NS), zero (Z), positive small (PS), positive medium (PM), positive big (PB), very small (VS), small (S), medium (M), small big (SB) and very big (MB).

a					$V_{L}$			
		NB	NM	NS	Z	PS	PM	PB
	**NB**	M	M	S	M	SB	SB	VB
	**NM**	S	M	S	S	M	SB	SB
	**NS**	VS	S	M	S	S	M	M
$V_{H}$	**Z**	SB	M	S	VS	S	S	M
	**PS**	SB	SB	M	M	VS	S	S
	**PM**	VB	SB	SB	SB	M	M	SB
	**PB**	VB	VB	VB	VB	SB	SB	M

**Table 4:** The subjects in the offline data collection experiments.

Subject No.	Age	Gender	Height	The Type of Disability
1	30	Female	160 cm	No
2	24	Female	160 cm	No
3	21	Male	170 cm	No
4	24	Male	170 cm	Left leg
5	24	Male	174 cm	Right leg
6	22	Female	158 cm	Left leg
7	23	Female	155 cm	Left and right leg

**Table 5:** The average relative distance when falling is detected.

	Fall Forward	Fall to Left	Fall to Right
The proposed fall detection algorithm	36 (cm)	26 (cm)	29 (cm)
Comparative fall detection algorithm	52 (cm)	37 (cm)	38 (cm)
